# Esmolol modulates inhibitory neurotransmission in the substantia gelatinosa of the spinal trigeminal nucleus of the rat

**DOI:** 10.1186/1471-2253-11-15

**Published:** 2011-09-05

**Authors:** Yutaka Yasui, Eiji Masaki, Fusao Kato

**Affiliations:** 1 Department of Anesthesiology, Jikei University School of Medicine, Minato-ku, Tokyo; 2 Division of Dent-oral Anesthesiology, Tohoku University Graduate School of Dentistry, Sendai, Miyagi, Japan; 3 Laboratory of Neurophysiology, Department of Neuroscience, Jikei University School of Medicine, Minato-ku, Tokyo 105-8461, Japan

## Abstract

**Background:**

β_1_-adrenaline receptor antagonists are often used to avoid circulatory complications during anesthesia in patients with cardiovascular diseases. Of these drugs, esmolol, a short-acting β antagonist, is also reported to exert antinociceptive and anesthetic sparing effects. This study was designed to identify the central mechanism underlying the antinociceptive effect of esmolol.

**Methods:**

Wistar rats (7-21 d, 17-50 g) were anesthetized with ketamine (100-150 mg/kg) or isoflurane (5%) and decapitated. Horizontal slices (400-μm thick) of the lower brainstem containing the substantia gelatinosa (SG) of the caudal part of the spinal trigeminal nucleus (Sp5c), in which the nociceptive primary afferents form the first intracranial synapses, were made with a vibrating slicer. The miniature inhibitory and excitatory postsynaptic currents (mIPSCs and mEPSCs, respectively) were simultaneously recorded from visually identified SG neurons of the Sp5c in the presence of tetrodotoxin (1 μM). Additionally, mIPSCs were recorded during pharmacological isolation of GABA- and glycine-mediated mIPSCs with kynurenic acid (1 mM).

**Results:**

Esmolol (500 μM) significantly and selectively increased the mIPSC frequency (to 214.2% ± 34.2% of the control, mean ± SEM, n = 35; *P *< 0.001), but not that of mEPSCs, without changing their amplitude. The increase in mIPSC frequency with esmolol was not affected by prior activation of β receptors with isoproterenol (100 μM) but it was significantly attenuated by removal of extracellular Ca^2+^.

**Conclusions:**

These data suggest that esmolol modulates inhibitory transmitter release in the Sp5c through a mechanism involving Ca^2+^-entry but in a β_1_-adrenoceptor-independent manner. The present results suggest that the facilitation of inhibitory transmitter release in the central nociceptive network underlies, at least in part, the antinociceptive effect of esmolol.

## Background

Antagonists of the β adrenoceptors are frequently used in patients with cardiovascular diseases to avoid circulatory complications during various types of operations requiring anesthesia. Esmolol, a short-acting β_1 _antagonist, was recently reported to exert antinociceptive and anesthetic-sparing effects in animals and human subjects. For example, esmolol inhibits nociceptive responses following formalin injection in rats [1], reduces anesthetic requirements for skin incision during propofol/N_2_O and morphine anesthesia in humans [2], reduces the volatile anesthetic requirement in patients receiving alfentanil [3], and reduces the intraoperative use of inhalation anesthetic and fentanyl as well as postoperative morphine consumption after perioperative administration esmolol [4] (a thorough summary of the analgesic and neuroprotective effects of β-blockers, including esmolol, can be found in a review by Kadoi and Saito, 2010) [5].

However, the mechanisms of such antinociceptive and anesthetic-sparing effects of esmolol remain largely unidentified. It is unlikely that such antinociceptive effects are simply attributable to the blockade of β_1 _receptors by esmolol because involvement of β_1 _receptors in the regulation of nociception in the spinal cord is limited or controversial [6-8]. In addition, a recent study indicated that esmolol, but not another potent β_1 _blocker, landiolol, blocks tetrodotoxin (TTX)-resistant Na channels involved in nociceptive signaling in the dorsal root ganglion [8], further suggesting that esmolol might exert its antinociceptive effect independent of its β receptor antagonism.

In addition to affecting the peripheral sensory systems, another possible mechanism of esmolol's antinociceptive effects is modulation of the activity of central networks underlying the transmission of nociceptive information, such as those in the spinal dorsal horn and in the spinal trigeminal nucleus [1,5,9]. In this study, we examined whether esmolol affects the activity of the central nociceptive network by recording spontaneously occurring synaptic currents in the substantia gelatinosa (SG) neurons of the caudal part of the spinal trigeminal nucleus (Sp5c). The Sp5c was chosen because it is the primary site of reception and modulation of thermosensitive and nociceptive signals arising from the cranio-orofacial regions, in which a variety of interneurons and projection neurons, together with a large number of bioactive substances, play essential roles in the modulation and integration of nociceptive information [10,11].

## Methods

### Brain slice preparation

The use of animals conformed to the Guiding Principles for the Care and Use of Animals in the Field of Physiological Sciences of the Physiological Society of Japan (1988) and was approved by the Animal Care Committee of the Jikei University School of Medicine, Tokyo, Japan. Wistar rats (7-21 days; weighing 17-50 g) of either sex were anesthetized by intraperitoneal ketamine (100-150 mg/kg) injection or brief isoflurane (5%) inhalation and decapitated immediately after the disappearance of the righting reflex. Using a vibration slice cutter (DTK-1000, Dosaka, Kyoto, Japan), two to three 400-μm thick horizontal brain slices through the Sp5c were made in ice-cold low-Ca^2+ ^and high-Mg^2+ ^artificial cerebrospinal fluid (ACSF) containing (in mM) NaCl 125, KCl 2.5, CaCl_2 _0.1, MgCl_2 _5.0, NaH_2_PO_4 _1.25, D-glucose 12.5, L-ascorbic acid 0.4, and NaHCO_3 _25 and saturated with 95% O_2 _+ 5% CO_2 _(pH = 7.4). The slices were incubated in "normal" ACSF (CaCl_2 _2 mM and MgCl_2 _1.3 mM) for 30-40 min at 37°C and then kept at room temperature until the recordings.

### Whole-cell recording

Two types of internal solutions were used [1]. A "CsCl-based" internal solution contained (in mM) 140 CsCl, 1 CaCl_2_, 2 MgATP, 1 EGTA, and 10 HEPES, pH 7.3, with CsOH. The estimated equilibrium potential of Cl^- ^with this internal solution was approximately 0 mV. This solution was used to record miniature inhibitory postsynaptic currents (mIPSCs) and miniature excitatory postsynaptic currents (mEPSCs), which appear independent of presynaptic action potentials. The frequencies of mIPSCs and mEPSCs reflect spontaneous and tonic transmitter release from inhibitory and excitatory presynaptic axon terminals, respectively. The mIPSCs were recorded in isolation at a holding potential of -70 mV in the presence of kynurenic acid (1 mM; an ionotropic glutamate receptor blocker; Sigma) and TTX (1 μM; a voltage-dependent Na^+ ^channel blocker; Alomone, Jerusalem, Israel), while mEPSCs were recorded in the presence of picrotoxin (100 μM; a GABA_A _and GABA_C _receptor blocker; Sigma), strychnine (1 μM; a glycine receptor blocker; Sigma), instead of kynurenic acid and TTX [2]. A "low-Cl" internal solution contained (in mM) 135 gluconic acid potassium, 0.1 CaCl_2_, 2 MgCl_2_, 2 MgATP, 0.3 NaGTP, 1 EGTA, and 10 HEPES, pH 7.3, with KOH. The estimated equilibrium potential of Cl^- ^with this internal solution was -90 mV. This internal solution was used to simultaneously record mEPSCs and mIPSCs from SG neurons in the Sp5c. In these experiments, the membrane potential was held around -40 mV, a value in between the reversal potentials of EPSCs and IPSCs, enabling simultaneous but separate recordings of inward (excitatory) and outward (inhibitory) postsynaptic currents. The tip resistance of the electrode with these solutions was 3-7 MΩ.

The slices were secured in a recording chamber (~0.5 ml volume) and continuously perfused with ACSF at a flow rate of 2-3 ml/min. Using infrared differential interference contrast optics or oblique illuminating systems combined with videomicroscopy (BX51; Olympus, Tokyo), the SG of Sp5c was identified as a lucent, rostrocaudally extending structure adjacent to the dark and opaque rostrocaudally running bundles of the trigeminal nerve at the lateral edge of the brainstem. The neurons located in the SG were visually identified, and all recordings were made from healthy-appearing neurons. Immediately (within 10 s) after the membrane rupture that established the whole-cell recording mode, we confirmed that the resting membrane potential was more polarized than -45 mV without current injection and, by rapidly manipulating the amplifier controls, that action potentials in response to positive current injection were overshooting. The cells without these properties were rare in our experimental conditions and were discarded when found. In the recordings with low-Cl internal solution, the resting potential and input membrane resistance were measured 5-10 min after the establishment of the whole-cell configuration. These values for the neurons recorded with the CsCl internal solution were not measured after stabilization because the resting membrane potential was almost 0 mV due to K channel blockade with Cs. The slices were perfused with "normal ACSF" during the search for and establishment of whole-cell configuration, and the data used for the analyses were sampled after at least a 10-min perfusion with specific ACSFs containing drugs for pharmacological isolation of the components of interest in each experiment. Only one neuron in a slice was recorded for pharmacological analyses. The nominally "Ca^2+ ^free" ACSF contained 3.3 mM MgCl_2 _and 0.2 mM EGTA (instead of 2 mM CaCl_2 _and 1.3 MgCl_2_) and was used to examine the role of extracellular Ca^2+^. The membrane current was recorded with an AxoPatch 200B (Axon Instruments). In a subset of the experiments, evoked IPSCs (eIPSCs), which were evoked by focal stimulation at a submaximal intensity (0.1 Hz; 0.08-0.5 mA; 100 μsec) with a bipolar concentric electrode placed within the Sp5C near the recording site (< 500 μm), were recorded together with spontaneous IPSCs (sIPSCs) in the absence of TTX and in the presence of kynurenic acid (1 mM).

In general, if a sole application of an antagonist exerts its effect by blocking a certain type of receptor, this effect should depend on how much these target receptors had been previously activated by endogenous ligands. Because esmolol markedly facilitated release at a much higher concentration than that at which it antagonizes β receptors, the β receptor agonist isoproterenol was pre-applied at 100 μM 10 min prior to esmolol in some of the experiments. Also, in a subset of cells, the effects of landiolol, which is another β blocker with a similar chemical structure to esmolol, were observed to examine whether landiolol also facilitates mIPSCs.

In general, an increase in the frequency of miniature postsynaptic events by a drug implies an effect on the presynaptic release mechanism [12]. Accordingly, the increase in mIPSC frequency, but not that of mEPSC, as described in the Results, might indicate that esmolol selectively affected GABAergic and/or glycinergic presynaptic terminals in the Sp5c.

We also analyzed the effect of extracellular Ca^2+ ^deprivation on esmolol modulation of mIPSC frequency to examine whether the effect of esmolol depends on the presence of extracellular Ca^2+ ^and its entry into the presynaptic terminals, which is the most critical step of transmitter release [13], to further identify the mechanism underlying the increase in mIPSC frequency with esmolol.

The signals were sampled with a PowerLab interface (AD Instruments) at 4 kHz. The series resistance was monitored but not compensated. The whole-cell capacitance was monitored and compensated. There were no apparent changes in the series resistance and whole-cell capacitance during the recordings for the neurons used in this study. The original traces in the figures and curve-fitting calculations were made with the Igor Pro graphic program (WaveMetrics). Postsynaptic currents were identified first automatically and then manually with visual identification of all events with IgorPro procedures written by F.K.

### Drugs

Esmolol (gift from Maruishi, Osaka), landiolol (gift from Ono Pharmaceutical, Osaka) and isoproterenol (Sigma) were dissolved in ACSF and applied via the perfusion line. Other compounds were purchased from Sigma or Nacalai Tesque (Kyoto, Japan). All recordings were conducted at room temperature (20 - 25°C).

### Statistics

The values are expressed as the mean ± standard error of the mean (SEM). Differences between the groups and between recordings before and during drug applications were examined with the nonparametric Mann-Whitney's U test for normalized values or Student paired t-test. Differences with probability (P) < 0.05 were considered significant. The concentration-response curves were drawn by fitting a sigmoidal curve to the data points at different drug concentrations using the curve-fitting function of Igor Pro (WaveMetrics).

## Results

**Figure 1:**
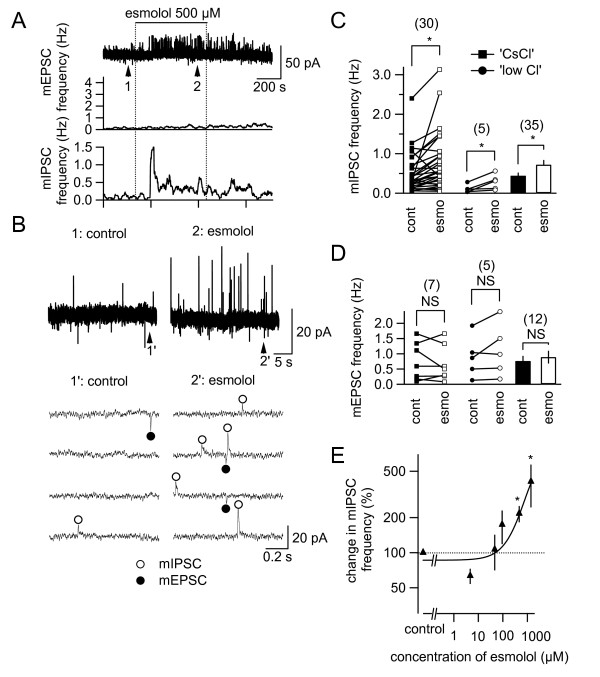
**Selective increase in miniature inhibitory postsynaptic current (mIPSC) frequency by esmolol in neurons of the caudal part of the spinal trigeminal nucleus (Sp5c)**. (A) Top: the membrane current recording of an Sp5c neuron at a holding potential of -40 mV. Esmolol was applied at the horizontal bar. Middle: the time course of the changes in miniature excitatory postsynaptic current (mEPSC) frequency with esmolol. Bottom: the time course of the changes in mIPSC frequency with esmolol. The abscissae of these graphs are identical (time; 5 min/div). (B) 1 and 2 are time-extended traces taken at the points 1 and 2 in the trace in A (top). mIPSCs and mEPSCs (outward and inward events, respectively) are marked with open circles above the traces and filled circles below the traces, respectively. (C) and (D) Summaries of the effects of esmolol on mIPSC and mEPSC frequency, respectively. "cont", mPSC frequency observed in 3-min control period before esmolol application (filled markers and bars); "esmo", those at 10-min application of esmolol (500 μM; open markers and bars). The numbers in parentheses indicate the number of neurons tested and were used for the statistics. Left, results of recordings with "CsCl-based" internal solution (squares) under pharmacological isolation of mIPSCs (C) and mEPSCs (D); center, results of recordings with "low-Cl^" ^internal solution in five cells (circles) in which mIPSCs (C) and mEPSCs (D) were simultaneously recorded (see Methods); right bars, pooled summaries based on the results with "CsCl" internal solution and "low-Cl" solution. *, *P *< 0.05; NS, not significantly different from pre-administration values (100%). Paired t-test. (E) Concentration-response relationship between esmolol and the changes in mIPSC frequency. The curve indicates the best-fit Hill equations for the data for esmolol. The number of neurons used to estimate the mean values and curve-fitting was 39 (control); 5 (5 μM); 5 (50 μM); 4 (100 μM); 35 (500 μM); 7 (1500 μM). *, *P *< 0.02; Mann-Whitney's U test; vs. control (no drug application). The horizontal broken line indicates the control values (100%).

**Figure 2:**
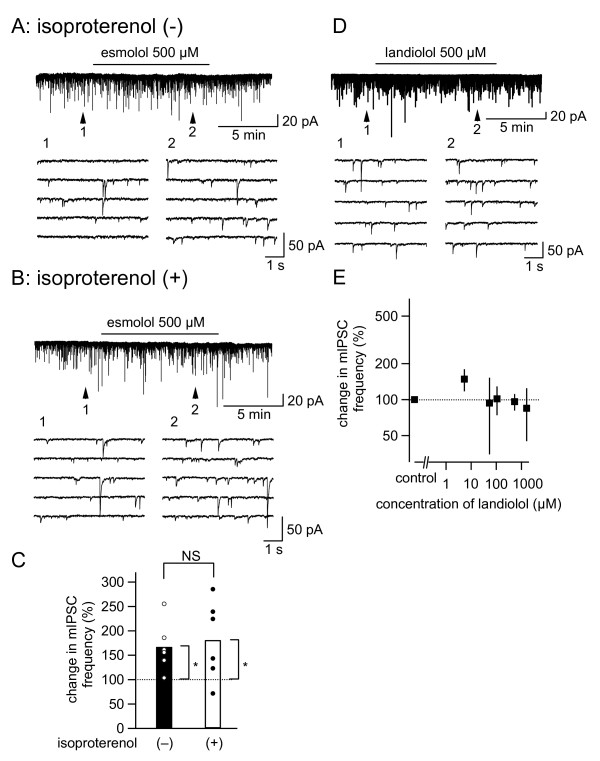
**Effects of β receptor agonists on the effect of esmolol and the effects of another beta antagonist, landiolol, on synaptic inhibitory transmission in the caudal part of the spinal trigeminal nucleus (Sp5c) neurons**. (A and B) Top: the membrane current recording of a neuron of the Sp5c in the absence (A) and presence (B) of 10-min prior administration of isoproterenol (100 μM). Recordings with "CsCl-based" internal solution. Esmolol was applied at the horizontal bar. Bottom of A and B: time-expanded continuous traces taken at points 1 and 2 in the top trace. (C) Summary of the effect of esmolol on mIPSC frequency in the absence (open circles; filled bar) and presence (filled circles; open bar) of isoproterenol. The bars show the average values. *, P < 0.05; Mann Whitney's U-test. NS, not significantly different; vs. pre-esmolol values. Each circle represents the data from one neuron (n = 6 neurons). (D) Top: the membrane current recording of a neuron of Sp5c using "CsCl-based" internal solution. Landiolol was applied at the horizontal bar. Bottom: time-expanded continuous traces taken at points 1 and 2 in the top trace. (E) Concentration-response relationship between landiolol and the changes in mIPSC frequency. The number of neurons used to estimate the mean values was 12 (control); 4 (5 μM); 4 (50 μM); 4 (100 μM); 10 (500 μM); 4 (1500 μM). Mann-Whitney's U test; vs. control (no drug application). The horizontal broken line indicates the control values (100%).

First, we analyzed the effects of esmolol on spontaneous action potential-independent postsynaptic currents (PSCs) recorded in the SG neurons of Sp5c in the presence of TTX. In the first series of experiments, we simultaneously recorded mEPSCs and mIPSCs using a low-Cl internal solution and by holding the membrane potential at -40 mV to examine whether esmolol exerts selective and differential effects on the excitatory and inhibitory transmissions in the same sets of neurons (Figure 1A). The resting membrane potential and input resistance for these neurons were -59.8 ± 0.5 mV and 182.0 ± 26.9 MΩ, respectively. Whereas esmolol markedly and significantly increased the mIPSC frequency (340.9% ± 174.3% (n = 5; *P *< 0.05) of the pre-esmolol value), the frequency of simultaneously recorded mEPSCs was not significantly affected by esmolol (135.2% ± 22.4% (n = 5; *P *= 0.18) of the pre-esmolol value; Figure 1A and 1B; summarized in Figure 1C; connected markers plot in the center; n = 5). This conclusion was also confirmed by the second series of experiments, in which mEPSCs and mIPSCs were recorded in separate sets of neurons under pharmacological isolation with picrotoxin and kynurenic acid, respectively, with a CsCl-containing internal solution at a holding potential of -70 mV. This series of experiments was performed because it allowed better identification of even smaller amplitude events. Whereas the frequency of mEPSCs recorded in isolation was not significantly affected by esmolol (118.3% ± 33.1% (n = 7; p = 0.57)), that of mIPSCs was significantly increased by esmolol to 185.9% ± 26.9% (n = 30; *P *< 0.001). When these responses are pooled, esmolol at 500 μM increased the frequency of mIPSCs to 214.2% ± 34.2% (n = 35; *P *< 0.001) of the pre-application value, without significantly affecting that of mEPSCs (125.3% ± 20.8% of the control; n = 12 neurons; Figure 1A and 1B). Esmolol (500 μM) did not exert a significant effect on the average mIPSC amplitude (before esmolol, 21.9 ± 4.2 pA; during esmolol, 21.8 ± 3.2 pA; *P *= 0.95; n = 6 neurons), suggesting limited effects on postsynaptic responsiveness. The increase in mIPSC frequency was dependent on the concentration of esmolol (Figure 2) and was significant at 500-1500 μM (*P *< 0.02; Figure 1). As the curve-fitting of a Sigmoidal curve resulted in an EC50 value larger than the whole estimated range, we concluded that the EC50 of esmolol might be larger than the concentration range examined (i.e., > 1500 μM; Figure 1D).

To directly examine whether the effects of esmolol on mIPSC frequency depend on prior activation of β receptors, we compared the effects of esmolol (500 μM) in the absence and presence (10-min pretreatment) of isoproterenol (100 μM). This concentration of isoproterenol gives rise to almost full activation of β receptors in brain slice experiments (14). The mIPSC frequency was not significantly affected by isoproterenol (112.9% ± 35.4% of the control, n = 6; *P *= 0.31). The increase in mIPSC frequency with esmolol in the absence of isoproterenol (*P *< 0.01; Figure 2C, open circles) was not significantly affected by the prior addition of isoproterenol (100 μM; *P *= 0.04; Figure 2C, filled circles). As a whole, the effect of esmolol in increasing mIPSC frequency was not apparently affected by prior activation of β receptors. In addition, we have examined effects of another selective β_1 _antagonist, landiolol (Figure 2D and 2E). Unlike esmolol (Figure 1E), landiolol, at concentrations of 5-1500 μM, did not significantly affect mIPSC frequency (Figure 2E); this finding further suggests that the effect of esmolol involves mechanisms other than β receptor blockade.

**Figure 3:**
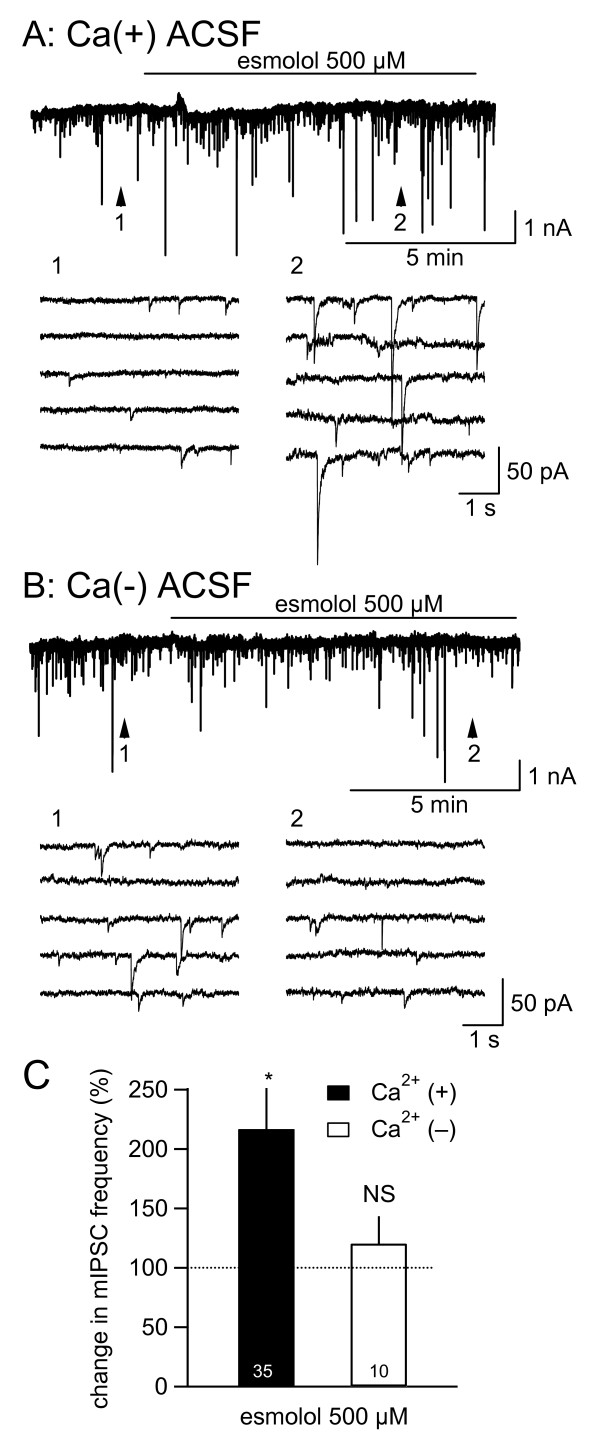
**The increase in miniature inhibitory postsynaptic current (mIPSC) frequency by esmolol was dependent on extracellular Ca^2+^**. (A and B) Top: the membrane current recording of a neuron of the caudal part of the spinal trigeminal nucleus in the presence (A) and in the absence (B) of extracellular Ca^2+^. Esmolol (500 μM) was applied at the horizontal bars. Recordings with "CsCl-based" internal solution. Bottom of A and B: time-expanded continuous traces taken at points 1 and 2 indicated in the top traces showing the control and the peak effects of drugs. (C) Summery of the effects of esmolol on mIPSC frequency. *, *P *< 0.001; Mann Whitney's U-test. NS, not significantly different; vs. pre-administration control values. The numbers in the bars indicate the number of neurons analyzed. The horizontal broken lines indicate the control values (100%).

The mIPSC frequency was not significantly affected by extracellular Ca^2+ ^deprivation (94.0% ± 15.7% of the control, n = 14). While esmolol significantly increased mIPSC frequency to 214.2% ± 34.2% of the control in the presence of 2 mM Ca^2+ ^(*P *< 0.001; Figure 3A, C; vs. pre-drug value (= 100%); Mann-Whitney U test), it only changed the frequency to 125.3% ± 20.8% (n = 10), which was not significantly different (P = 0.42) from the pre-esmolol value, in the absence of extracellular Ca^2+ ^(Figure 3B, C).

**Figure 4:**
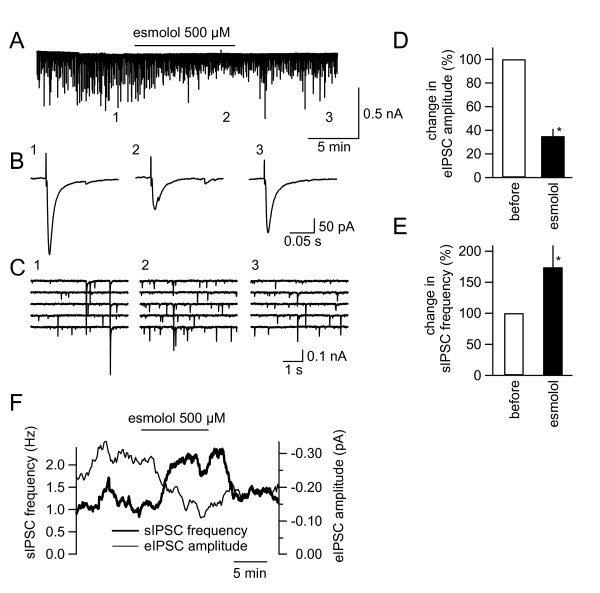
**The increase in spontaneous inhibitory postsynaptic current (sIPSC) frequency by esmolol in neurons of the caudal part of the spinal trigeminal nucleus (Sp5c)**. (A) The membrane current recording of an Sp5c neuron. Esmolol was applied at the horizontal bar. (B) Traces showing evoked IPSC (eIPSC) waveforms (average of eight consecutive traces) evoked by stimulation before (1; left), during (2; middle) and after (3; right) esmolol application in the Sp5c. 1-3 were sampled at points 1-3 in A. (C) 1-3 are time-extended traces taken at points 1-3 in the trace in A. (D) and (E) Summary of the effects of esmolol on eIPSC amplitude and sIPSC frequency, respectively. *, P < 0.05; Mann-Whitney's U-test. (F) The time course of the changes with esmolol in sIPSC frequency (thick line) and eIPSC amplitude (thin line) that were simultaneously recorded. The abscissae of these graphs are identical (time).

We then analyzed the effects of esmolol on sIPSCs and eIPSCs recorded in the SG neurons of Sp5c in the absence of TTX. Esmolol (500 μM) significantly increased sIPSC frequency (to 174 ± 35.4% of the control; n = 4) without significantly affecting the amplitude of sIPSCs (before esmolol, 47.1 ± 9.1 pA; during esmolol, 43.0 ± 8.3 pA: Figure 4A, C, E and 4F). In contrast to the increase in the frequency of sIPSCs, esmolol significantly decreased eIPSC amplitude to 35 ± 6.2% of the control (Figure 4B and 4D). This decrease in eIPSC amplitude was inversely related to the increase in sIPSC frequency in each neuron (e.g. Figure 4D).

## Discussion

By recording postsynaptic currents in the visually identified SG neurons in the Sp5c, we found that esmolol modulates spontaneous inhibitory transmission. The major findings are that 1) esmolol selectively increases the frequency of mIPSCs and sIPSCs without affecting that of mEPSCs, 2) esmolol decreases the amplitude of eIPSCs without affecting that of either mIPSCs or sIPSCs in the SG neurons of Sp5c, 3) the increase in mIPSC frequency with esmolol requires extracellular Ca^2+^, and 4) this increase in mIPSC frequency with esmolol does not require prior activation of β receptors. Because the Sp5c is the primary center of integration and modulation of primary afferent fibers carrying nociceptive and thermosensitive information arising from orofacial regions (15) and because inhibitory transmission is the primary mechanism determining the "gating" of nociceptive information [16], these findings might explain part of the basis for the analgesic effect of esmolol described to date [1-3].

### Mechanism of release facilitation

The mechanism through which esmolol facilitates the release of inhibitory transmitters remains unidentified. Interestingly, in the present preparation, extracellular Ca^2+ ^deprivation did not significantly affect the basal frequency of mIPSCs, suggesting that a large part of the spontaneous release occurred through mechanisms independent of Ca^2+ ^entry, which is a phenomenon commonly observed in the spinal SG [17] and the nucleus of the solitary tract [12]. The present result indicates that the increase in mIPSC frequency by esmolol does not involve facilitation of such Ca^2+ ^entry-independent release. It is therefore likely that esmolol promoted the process upstream to vesicle fusion [13,18]. The present effects of esmolol are reminiscent of EPSC potentiation by noradrenaline through an identified mechanism not involving α and β adrenoceptors in the chick ciliary ganglion synapses [19]. Yao [19] attributed this effect of noradrenaline to an enhanced vesicle fusion probability resulting from an increased Ca^2+ ^sensitivity of the exocytotic process. Whether a similar mechanism underlies the effect of esmolol in the SG of Sp5c remains undetermined.

Interestingly, esmolol exerted opposing effects on the spontaneous and evoked IPSCs. Such contrasting effects are reminiscent of the effect of BDNF on GABA and glycine release in the spinal dorsal horn [20]. The precise cellular mechanisms underlying these opposing effects of esmolol remain to be examined in future studies.

### Selective facilitation of inhibitory transmission

Another interesting feature of the release facilitation by esmolol was its selective effect on inhibitory transmission. The most straightforward interpretation of this selective facilitation is that the molecular mechanism underlying this effect of esmolol is exclusively expressed at GABAergic and/or glycinergic terminals. This is not surprising because, for example, release facilitation by activation of presynaptic Ca^2+^-permeable P2X receptors in the spinal dorsal horn occurs exclusively at glycinergic terminals [17] and that in the nucleus of the solitary tract [12] occurs only at glutamatergic terminals, owing to selective expression of these receptors at glutamatergic terminals. We did not pharmacologically identify whether these IPSCs were mediated by GABA_A _receptors, glycine receptors, or both. In the spinal dorsal horn of young animals, as used in this study, glycine-only, GABA-only, and mixed GABA/glycine IPSCs were recorded [21,22], suggesting that the mIPSCs recorded in this study are also composed of postsynaptic currents mediated by these multiple inhibitory transmitters.

Such selective facilitation of inhibitory transmission was also reported with nocistatin, a neuropeptide shown to be involved in the development and/or modulation of hyperalgesia and allodynia. In the superficial layer of the spinal cord dorsal horn of rats, nocistatin selectively inhibits inhibitory, but not excitatory, transmission, due to selective expression of its receptors in the inhibitory interneurons in the SG [23]. Likewise, a cannabinoid agonist selectively decreases mIPSC frequency, but not that of mEPSCs, in rat cerebellar Purkinje neurons, presumably due to the distinct roles of intracellular Ca^2+ ^in generation of action potential-independent release between the glutamatergic and GABAergic terminals [24]. These examples suggest that the molecular mechanism mediating esmolol's effects is expressed selectively at inhibitory terminals. Because inhibitory transmission in the SG plays essential roles [17] in avoiding transmission of excessive nociceptive signals to the higher centers and motor systems involved in the withdrawal reflex [22], such selective facilitation of inhibitory transmission should prominently reduce nociceptive transmission, thus resulting in an analgesic effect.

### Molecular mechanism of esmolol effects and clinical implication

Another interesting but unexpected feature of the facilitory effect of esmolol on mIPSC frequency found in this study was that esmolol exerted this effect through an unidentified mechanism not involving β_1_-adrenoceptor blockade. The following three lines of evidence argue against an involvement of β_1_-adrenoceptor blockade in the nociceptive effect of esmolol. First, the EC50 for the increase in mIPSC frequency (> 1500 μM) was much larger than that reported for β-receptor blockade (~10 μM). Second, landiolol, another short-acting β_1_-blocker, did not exert detectable changes on mIPSC frequency in the present preparation and experimental conditions. Third, this effect of esmolol was not affected by prior activation of β-receptors with isoproterenol. In addition, the effect of activating β_1 _receptors on nociceptive signaling in the spinal dorsal horn [25] remains controversial [6,7]. At this moment, we have no candidate molecule that might mediate such non-adrenergic analgesic effects of esmolol. As the facilitory effect of esmolol was observed at a relatively high concentration (> 500 μM), it is possible that esmolol also blocked β_2 _receptors because of its small difference in binding affinity (34-fold) [26]. However, it has been reported that an activation, not a blockade, of β_2 _receptors has an antinociceptive effect in animals with neuropathic pain [27], making the involvement of β_2 _receptor blockade by esmolol in mIPSC facilitation unlikely.

The mechanism underlying the effects described in this study might be distinct from, but could be cooperative with, the non-adrenergic inhibition of TTX-resistant Na current with esmolol [8]. Notably, for technical reasons, the present data were obtained in 7- to 21-day-old rats (most were 10-21 days). To our knowledge, an analgesic effect of esmolol in immature human subjects or animals has never been examined or described. As the inhibitory synaptic organization in the spinal cord dorsal horn [21] undergoes postnatal modification, it will be important in the future to confirm that such effects of esmolol on spontaneous inhibitory transmission could be reproduced in more mature animals.

## Conclusion

This study indicates that esmolol modulates inhibitory transmitter release in Sp5c neurons through a mechanism involving Ca^2+ ^entry and in a β_1_-adrenoceptor-independent manner. Such release facilitation of inhibitory transmitters might underlie, at least in part, the antinociceptive effect of esmolol. Identification of the molecular target of such an effect of esmolol could lead to better strategies for the management of operative and persistent pain.

## Competing interests

The authors declare that they have no competing interests.

## Authors' contributions

YY participated in the data acquisition, data analysis, interpretation and drafting of the manuscript. FK participated in the study conception, study design, data analysis, interpretation and drafting of the manuscript. EM participated in the study conception, study design and data acquisition. All authors read and approved the final manuscript.

## Pre-publication history

The pre-publication history for this paper can be accessed here:

http://www.biomedcentral.com/1471-2253/11/15/prepub
